# Evaluation of the iFalcon^TM^V100 rebound tonometer in ex-vivo rabbit eyes

**DOI:** 10.1186/s12917-026-05355-5

**Published:** 2026-03-05

**Authors:** Yunhao Su, Jing Jin, Hao Cheng, Ji Fu

**Affiliations:** The school of Mechanical Engineering, Zhejiang Sci-Tech University, Hangzhou, China

**Keywords:** Intraocular pressure, Rebound tonometry, Ex vivo, Implantable pressure sensor, Tonometry accuracy

## Abstract

Accurate intraocular pressure (IOP) measurement is fundamental for both clinical ophthalmology and experimental research. Study evaluated the performance of iFalcon^TM^V100 rebound tonometer in four enucleated rabbit eyes. We employed a stepwise pressure-increasing protocol to generate a physiologically relevant IOP range (5–55 mmHg). At each target pressure, measurements were taken concurrently with the tonometer (using the rabbit-specific mode) and the reference sensor. We used linear regression and Bland-Altman analysis to assess agreement across the entire dataset, supplemented by a segmented analysis of normal and elevated IOP ranges. Overall linear regression revealed a strong correlation between the iFalcon^TM^V100 Vet and the reference sensor(R² = 0.9712, *p* < 0.001); however, interval-dependent bias was present. The positive intercept (0.8951) suggested a tendency for the tonometer to overestimate IOP at low measurement range, while slope < 1 suggested systematic underestimation at higher pressures. Bland-Altman analysis further confirmed this systematic underestimation bias at central corneal position. The iFalcon^TM^V100 rebound tonometer showed strong agreement with direct sensor measurements across a broad pressure range (5–55 mmHg) in an ex vivo rabbit model, supporting its potential utility for IOP assessment in this setting, with limitations noted in extreme pressure ranges.

## Introduction

Accurate IOP measurement bridges basic ophthalmic research and clinical translation. It is crucial for diagnosing and monitoring diseases like glaucoma [1] and for preventing vision loss from elevated IOP. Consequently, the reliability of IOP data underpins both the validity of research findings and the safety of clinical practice [2, 3].

Rabbit eyes have emerged as a core model for IOP-related studies due to their high similarity to human eyes in terms of corneal biomechanical properties [4, 5] and anterior chamber anatomical structure, along with advantages including controllable breeding costs and ease of standardizing experimental procedures [6]. However, obtaining accurate and reproducible IOP measurements in rabbits presents specific challenges. Studies have demonstrated that IOP readings in this species are highly sensitive to the type of tonometer used and to experimental conditions, including methods of physical restraint or anesthesia, even in clinically normal eyes [7]. This need for rigorous, condition-aware validation extends beyond rabbits to other key animal models in ophthalmic research, such as chickens and sheep, which are vital for both comparative studies and veterinary clinical science [8–13]. Therefore, rigorous validation of any tonometric device for its intended species and under defined conditions is essential for the reliable interpretation of experimental data. Traditional gold-standard Goldmann Applanation Tonometer (GAT) requires operator expertise and topical corneal anesthesia [12]. It is not only difficult to adapt to the physiological structure of rabbit eyes but also unable to meet the needs of dynamic monitoring or high-throughput experiments [14]. While early commercial rebound tonometers of the iCare series achieved portability and non-invasiveness, their reliance on a generalized linear algorithm may not adequately accommodate variations in species-specific corneal biomechanics [15–17]. This poses a particular challenge in extreme IOP ranges, where accurate data are critical yet difficult to obtain, highlighting the need for more adaptable tonometry solutions [18]. 

The emergence of species-specific rebound tonometers has been a key technological breakthrough in recent years, with its core innovation lying in algorithms tailored to species-specific ocular biomechanics [19, 20]. Initial validation in enucleated rabbit eyes confirmed that the TonoVet^®^ Plus tonometer performs reliably across a wide pressure range, although it systematically overestimates IOP at lower levels and slightly underestimates it at higher pressures [21]. Further evaluation in canine eyes, with IOP stratified into normal and elevated rYunhao Su and Hao Chenganges, demonstrated that while both the TonoVet^®^ Plus and its predecessor exhibit clinically acceptable agreement with manometric reference values within the normal IOP range, a systematic compression effect persists in the low-to-normal pressure segment, indicating that current algorithms still tend to bias measurements toward the midrange [22]. This consistent compression underscores the need for further refinement of species-specific tonometric algorithms, especially for the accurate assessment of physiologically and clinically relevant IOP levels. This persistent compression highlights the need for further refinement of species-specific algorithms to improve accuracy in clinically important pressure ranges.

As a new device in this field, the iFalcon^TM^V100 rebound tonometer innovatively incorporates Hertzian contact theory to construct a nonlinear physical model [23]. Theoretically, analyzing the dynamic attenuation curve of the probe post-corneal impact allows for a more accurate inversion of IOP values, particularly in species with specific corneal biomechanical properties like rabbits, and could potentially mitigate the measurement biases of traditional devices. Nevertheless, the practical effectiveness of any algorithm optimization requires verification through standardized experiments. The applicability of this nonlinear model to rabbit corneas and its actual measurement accuracy have not yet been rigorously validated, especially the measurement stability in the low IOP range and the reading linearity in the high IOP range, which directly affect the device’s applicability in studies involving glaucoma models, corneal injury models, and other related research.

This study aims to fill this gap by establishing an absolute IOP reference standard using an implantable pressure sensor, to systematically validate the measurement accuracy of the iFalcon^TM^V100 rebound tonometer within the range of 5–55 mmHg. We hypothesized that the iFalcon^TM^V100 would exhibit strong agreement with the reference sensor across the entire IOP range; however, we anticipated that it’s accuracy would vary with IOP level, attributable to inherent limitations in the device’s nonlinear calibration algorithm and constraints associated with the ex vivo experimental model.

## Materials and methods

### Participants

Four healthy rabbit eyes (both eyes from two rabbits) used in this study were purchased from a local animal experimental center (Lifutai Bio Co.,Ltd.). The sample size was chosen in accordance with common practice in similar ex vivo validation studies for ophthalmic tonometers, which often utilize a limited number of eyes to establish proof-of-concept and assess fundamental agreement under controlled conditions [24]. The implications of this limited sample size on the generalizability of the findings are explicitly addressed as a key limitation in the Discussion. Rabbit eyes were obtained postmortem. The rabbits had been euthanized as part of other independent research projects; no animals were sacrificed specifically for this study, and no live animals were involved. Only eyes with no known history of ocular disease or intervention were selected post-euthanasia. All procedures were performed by a qualified veterinarian. First, overdose anesthesia was induced by intravenous administration of propofol (10 mg/kg) via the marginal ear vein. Immediately following anesthetic induction, euthanasia was carried out by exsanguination via the carotid artery. Bilateral enucleation was then performed under aseptic conditions to obtain intact globes. Immediately after enucleation, the eyes were rinsed with sterile normal saline. Subsequently, a board-certified veterinary ophthalmologist examined all eyes via slit-lamp biomicroscopy to confirm gross corneal clarity, anterior chamber depth, and lens position. No macroscopic structural abnormalities (e.g., corneal scarring, synechiae, or significant opacities) were observed. Following this examination, the globes were placed in a sterile, sealed container on ice, transported to the experimental site, and all subsequent experiments were completed within 12 h post-enucleation. Notably, this examination was designed to exclude major defects without fluorescein staining and therefore could not rule out subtle surface irregularities such as superficial corneal ulcers. The potential impact of this limitation is addressed in the Discussion.

### IOP measurement

**Fig. 1:**
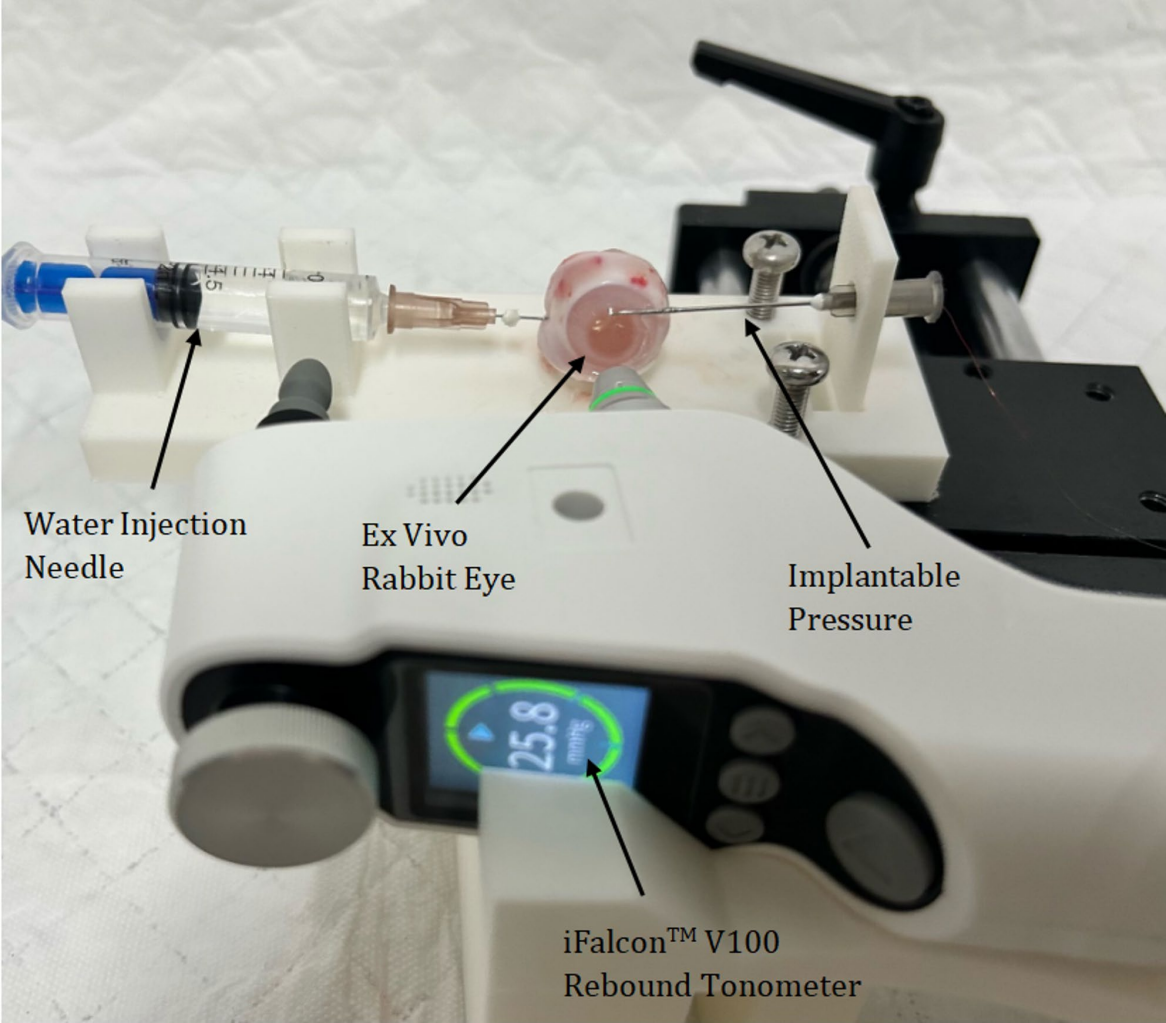
Experimental platform setup

We used a custom-designed three-dimensional (3D) fixation platform to securely position all key components: the rebound tonometer, ex vivo rabbit eye, implantable pressure sensor(ZXPAYZ040B, Beijing Smart Sensor Technology Co., Ltd.), and water pressure syringe (Fig. 1).The ex vivo rabbit eye was secured at the center of a custom-designed 3D-printed platform. Syringe holder and sensor holder were mounted on the left and right sides respectively. This configuration provided stable support for the water pressure syringe and the implantable pressure sensor, minimizing displacement or vibration during measurements. The water pressure syringe was inserted into the anterior chamber via a paracentesis at the corneal limbus [25, 26], while the implantable pressure sensor was introduced into the vitreous cavity through a scleral puncture on the contralateral side [27–29]. All puncture sites were sealed with cyanoacrylate tissue adhesive. Pressure tests confirmed no fluid leakage, ensuring measurement accuracy. In addition, a specialized holder was attached to the platform to secure the iFalcon^TM^V100 rebound tonometer—this setup guaranteed that the tonometer’s probe kept a consistent distance from the central cornea and remained vertically aligned with it.

We employed a stepwise protocol to precisely control IOP using a water-filled syringe system, incrementally increasing pressure from a baseline of 5 mmHg to a maximum of 55 mmHg. Measurements commenced only after the pressure remained stable at each preset level for 10 s, as confirmed by the real-time reading from the implantable pressure sensor. The iFalcon^TM^V100 rebound tonometer, set to its rabbit-specific operational mode, was used to record 6 successive readings at each pressure point. The device features built-in modes for multiple animal species including rabbit, cat, horse, dog, monkey, and rodent. We selected the rabbit mode, which the manufacturer indicates has been calibrated for rabbit intraocular pressure(IOP). In accordance with the manufacturer’s guidelines, the probe tip was positioned approximately 4–8 mm from the corneal apex. The fluid infused into the anterior chamber was sterile physiological saline at room temperature. All measurements were conducted in a laboratory environment at room temperature. New, disposable probe was used for each eye before the experiment to prevent cross-contamination.

### Statistical analysis

All statistical analyses were conducted using Python 3.12. Linear regression [30] was applied to evaluate the correlation between IOP measurements taken with the iFalcon™ V100 tonometer and the implantable pressure sensor. The strength of the linear relationship was quantified with the regression slope, intercept, and coefficient of determination (R²). Subsequently, the data were divided into two intervals: normal IOP (5–25 mmHg) and high IOP (26–55 mmHg), and segmented linear regression analyses were performed for each interval to assess the correlation characteristics of the iFalcon^TM^V100 tonometer across different pressure ranges. Agreement between the two methods was evaluated using Bland-Altman analysis [31–33], both across the entire range (5–55 mmHg) and within each pressure interval. Each Bland-Altman analysis yielded the mean bias (iFalcon^TM^V100 – sensor) and the 95% limits of agreement, which together describe the measurement agreement and systematic bias of the iFalcon^TM^V100 tonometer. All figures were generated using the matplotlib library (version 3.8.2).

## Results

**Fig. 2:**
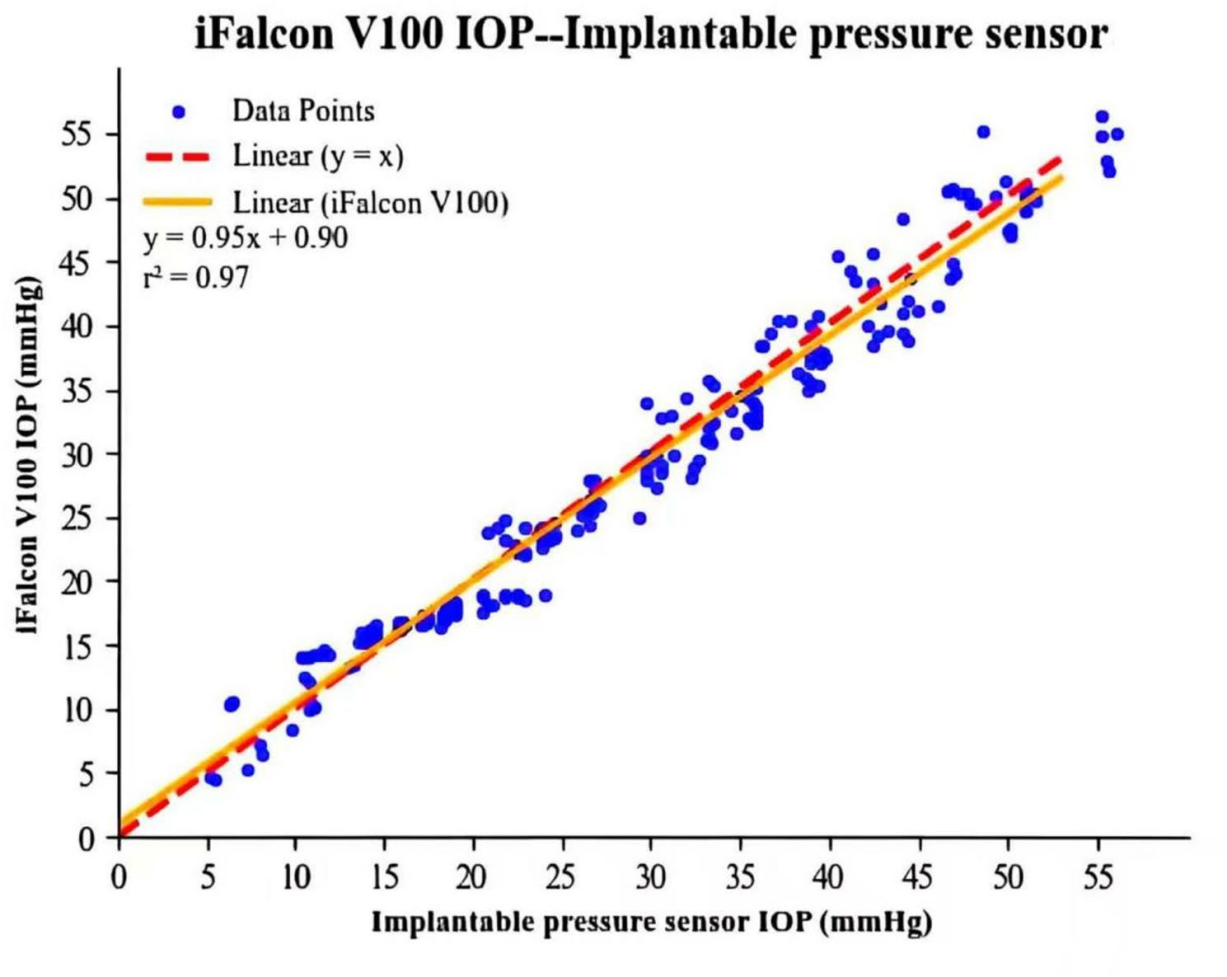
Linear regression analysis of the rebound tonometer (iFalcon^TM^V100) versus the implantable pressure sensor

Global linear regression analysis (Fig. 2) revealed a strong linear correlation between the measurements of the iFalcon^TM^V100 rebound tonometer and the implantable pressure sensor across the 5–55 mmHg range in all four eyes (regression equation: y = 0.95x + 0.90; r² = 0.97, *p* < 0.001). This result indicates that the iFalcon^TM^V100 effectively tracks changes in true IOP. However, regression equation (slope = 0.95, intercept = + 0.90 mmHg) reveals a pattern of slight systematic underestimation at higher pressures and a tendency toward overestimation at lower pressures, particularly near 0 mmHg. Consequently, the accuracy of the iFalcon^TM^V100 is range-dependent, with both the direction and magnitude of its measurement bias varying according to the underlying true IOP level.

**Fig. 3:**
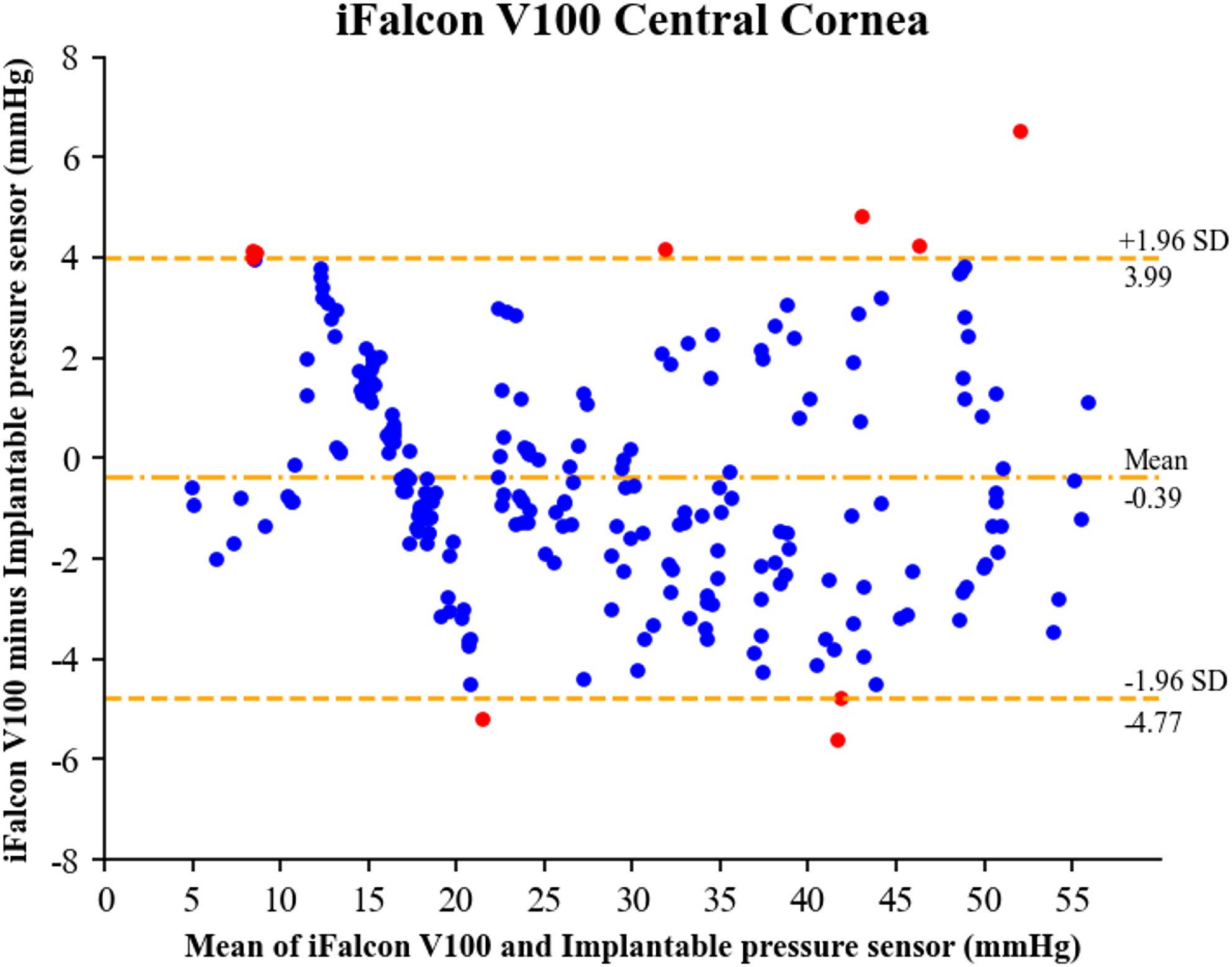
Bland-Altman analysis of the rebound tonometer (iFalcon^TM^V100) versus the implantable pressure sensor

Global Bland-Altman analysis (Fig. 3) further quantified the agreement between the two methods. The iFalcon^TM^V100 readings showed a mean bias of −0.39 mmHg relative to the sensor, consistent with the underestimation trend indicated by linear regression. The 95% limits of agreement (LoA) spanned from − 4.77 to + 3.99 mmHg. Notably, within the extremely low-pressure range of 5–10 mmHg, multiple measurement attempts frequently failed, requiring repetition to obtain valid data. These measurement failures were absent at pressures of 10 mmHg and above.

**Figs. 4:**
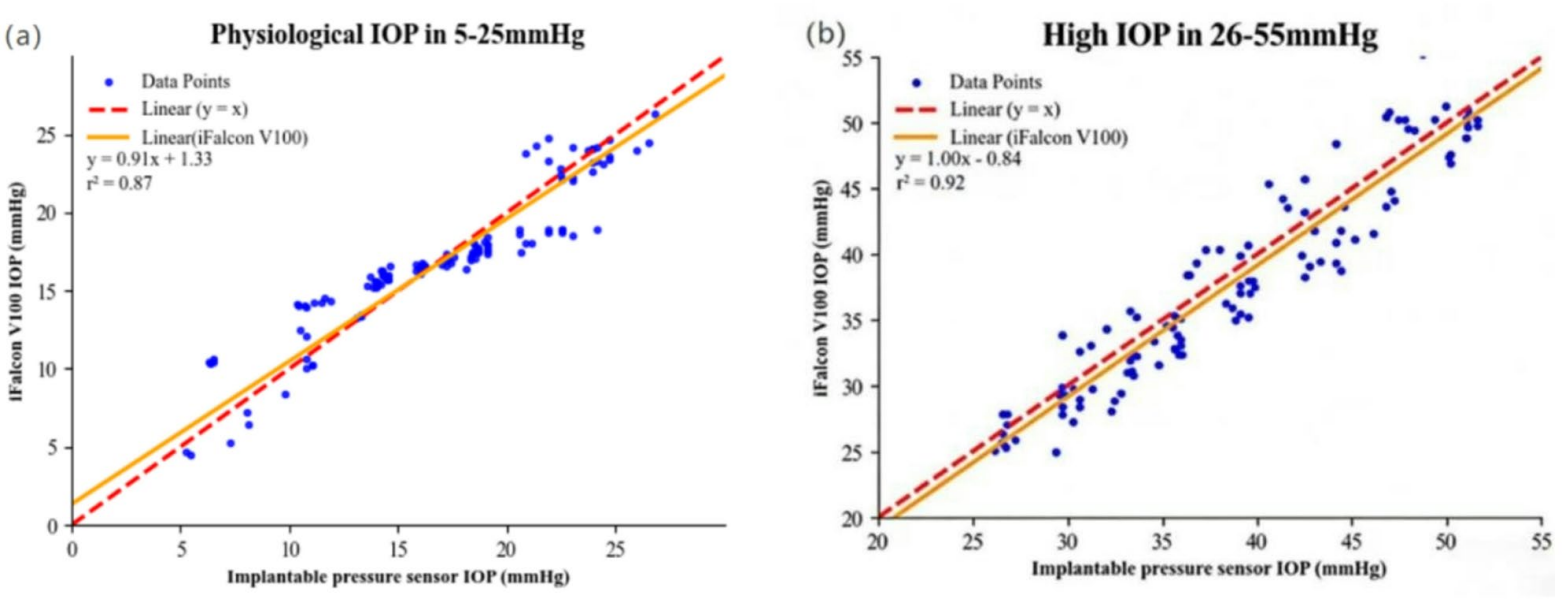
Linear regression between the iFalcon^TM^V100 versus the implantable pressure sensor across the normal and high IOP ranges. Panel (**a**) represents the normal IOP range (5–25 mmHg), panel (**b**) represents the high IOP range (26–55 mmHg)

Segmented linear regression analysis (Fig. 4) examined the correlation between IOP measurements from the iFalcon^TM^V100 rebound tonometer and the implantable pressure sensor across distinct intervals. In the normal IOP interval (5–25 mmHg), the segmented linear regression equation was y = 0.91x + 1.33 (R² = 0.87). Based on the standard range of 15–25 mmHg reported in the literature, the broader interval of 5–25 mmHg was adopted for analysis to account for potential physiological fluctuations [34]. The slope less than 1 and the positive intercept indicated that the iFalcon^TM^V100 tonometer exhibited systematic overestimation of IOP within this range, and this overestimation tendency became more pronounced as the true IOP decreased.In the high IOP interval (26–55 mmHg), the segmented linear regression equation was y = 1.00x – 0.84 (R² = 0.92). The slope of 0.9996 was presented as 1.00. The slope close to 1 reflected good overall agreement between the iFalcon^TM^V100 measurements and the implantable sensor readings in this interval, while the negative intercept suggested a slight systematic underestimation of IOP by the iFalcon^TM^V100 tonometer at relatively higher pressure levels. These results demonstrate that although the iFalcon^TM^V100 rebound tonometer effectively tracks IOP across the tested range, its accuracy exhibits distinct interval dependence—with the direction and magnitude of measurement bias varying with the level of true IOP.

**Figs. 5:**
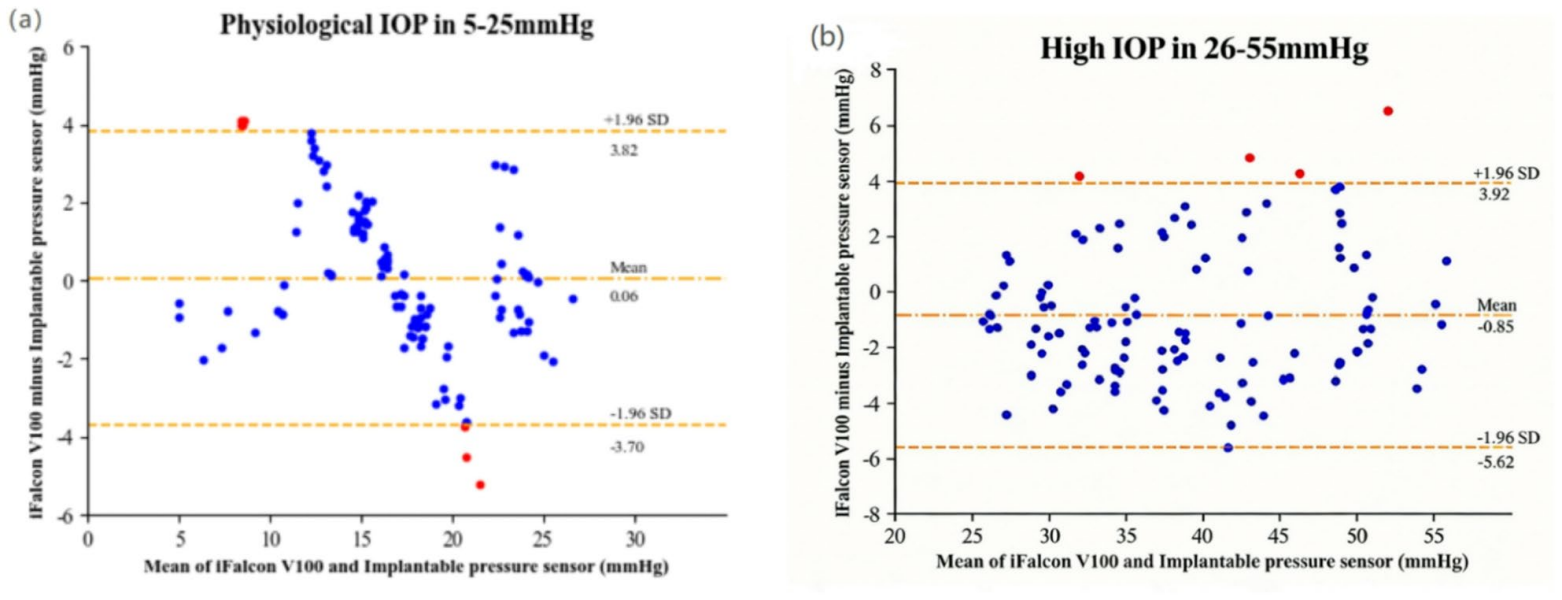
Bland-Altman analysis of the iFalcon^TM^V100 versus an implantable pressure sensor across the normal and high IOP Ranges. Panel (**a**) represents the normal IOP range (5–25 mmHg), panel (**b**) represents the high IOP range (26–55 mmHg)

Segmented Bland-Altman analysis (Fig. 5) further revealed the error profile of the iFalcon^TM^V100 rebound tonometer across different IOP intervals. In the normal IOP range, the mean bias was 0.06 mmHg, with 95% LoA ranging from − 3.7 to + 3.82 mmHg. This suggests no substantial systematic bias on average within this interval. However, the random error (width of the 95% LoA) was relatively wide, approximately 7.52 mmHg. In the high IOP interval, the mean bias shifted to −0.85 mmHg, with the 95% LoA widening to −5.62 to + 3.92 mmHg. These results demonstrate a distinct trend of systematic underestimation in this interval, in parallel with a further increase in random error to approximately 9.54 mmHg.Notably, in the extremely low IOP range (5–10 mmHg), measurement attempts frequently failed, necessitating repeated attempts to obtain stable, valid readings. In contrast, no measurement difficulties were encountered at IOPs of 10 mmHg or higher, indicating that the reading stability of the device in the extremely low IOP range still needs improvement. It is important to note that this limitation is not unique to the iFalcon^TM^V100 but is consistent with the documented challenges of rebound tonometry at low ocular tension in animal models [21, 35]. These findings indicate that the iFalcon^TM^V100 provides reliable and essentially unbiased measurements within the physiologically critical normal IOP range. Furthermore, the device effectively tracks elevated IOPs, despite a well-defined and acceptable systematic deviation. Its performance limitation is primarily confined to the clinically less frequent very low pressure range.

## Discussions

The lack of experimental validation for the iFalcon^TM^V100’s nonlinear model in rabbit eyes, as noted above, represents a critical knowledge gap. Therefore, this study evaluates the iFalcon^TM^V100 rebound tonometer in an ex vivo rabbit eye model, with a specific focus on directly assessing the practical performance of the device’s foundational nonlinear physical model, which is based on Hertzian contact theory. The model operates by fitting the dynamic decay curve of the probe following corneal impact, thereby providing a more accurate inversion of IOP values. This approach overcomes the measurement deviations inherent in traditional devices that rely on linear models. Experimental results validated the device’s effectiveness: within the IOP range of 5–55 mmHg, the iFalcon^TM^V100 exhibited a strong linear correlation with the implantable pressure sensor (R² = 0.97), with only minor systematic errors observed at the two extremes of this pressure range. This performance aligns with previously reported validation data for the TonoVet^®^ Plus (R² = 0.99, 5–70 mmHg). Both devices demonstrated good agreement within the physiological pressure range (10–30 mmHg), a performance that may be suitable for basic experimental applications, though clinical applicability requires further in vivo validation [21]. 

Error analysis revealed notable commonalities between the iFalcon^TM^V100 and TonoVet^®^ Plus in the ex vivo rabbit eye model. The 95% LoA were ± 4.38 mmHg for the iFalcon^TM^V100 and approximately ± 5 mmHg for the TonoVet^®^ Plus (estimated from figures, not directly provided in the referenced documents). Both ranges are consistent with the typical variation observed in ex vivo models. The two devices also showed similar limitations at pressure extremes. The iFalcon^TM^V100 exhibited multiple measurement failures in the 5–10 mmHg range, whereas the TonoVet^®^ Plus encountered difficulties at pressures below 10 mmHg. The consistency of these issues across devices suggests that they may stem, at least in part, from fundamental limitations of the rebound tonometry principle when at low IOP. Previous studies have hypothesized that at low corneal tension, the deceleration profile of the probe becomes less distinct and more susceptible to measurement noise, leading to increased variability and occasional failure to obtain valid readings [21, 22]. Thus, the instability we observed in the 5–10 mmHg range likely reflects a common constraint of the rebound method under these conditions, rather than a defect unique to either device. Moreover, mild underestimation was generally observed in the high-pressure range (slope = 0.95 for the iFalcon^TM^V100, slope = 0.90 for TonoVet^®^ Plus) [6]. These commonalities suggest that, in addition to differences in algorithms, errors may collectively arise from the limitations of the ex vivo model itself: the elastic modulus of the rabbit corneal tissue decreases within 12 h after death; and although the puncture sites were sealed with tissue adhesive, negligible fluid leakage may still occur [36, 37]. However, this remains a hypothesis, as the present study did not experimentally distinguish between device-related and model-related error sources. Therefore, evaluating rebound tonometer performance requires a comprehensive consideration of both device-specific technical parameters and the inherent characteristics of the experimental model. Instead of judging a device solely based on numerical deviations, its applicability should be assessed in conjunction with the application scenario (e.g., routine physiological measurement or monitoring of extreme pathological conditions).

Further detailed analysis of the iFalcon^TM^V100’s errors reveals a distinct range dependence of its measurement biases. Segmented linear regression analysis indicates that in the normal IOP interval, the device exhibits systematic overestimation of IOP (intercept = + 1.33 mmHg), and this overestimation trend becomes more pronounced as IOP decreases. The segmented regression equation (y = 0.91x + 1.33) allows quantification of this overestimation. For instance, at a true IOP of 5 mmHg, the predicted measured value is 5.88 mmHg, corresponding to a relative overestimation of 17.6%. At 10 mmHg, the predicted measured value becomes 10.43 mmHg, with a relative overestimation of 4.3%. From an experimental standpoint, such overestimation is generally acceptable in most basic rabbit ophthalmic studies. For instance, in studies investigating glaucoma pathogenesis or evaluating hypotensive therapies (where the primary endpoint is IOP elevation above 25 mmHg [6]), the mild overestimation in the normal range does not interfere with distinguishing between physiological and pathological IOP or assessing treatment efficacy. In these contexts, the observed deviation in the normal range is unlikely to compromise the distinction between physiological and pathological IOP or the assessment of therapeutic efficacy. However, in research specifically targeting low IOP phenomena, such as studies on corneal endothelial viability under hypotony or wound healing in low-pressure models—this systematic bias may introduce non-negligible error. In such specialized applications, we recommend applying the derived regression correction (corrected IOP = [measured IOP – 1.33]/0.91) to mitigate the overestimation effect. Clinically, if the iFalcon^TM^V100 is employed in vivo—for instance, in preclinical device validation—the observed overestimation is unlikely to lead to misclassification of hypotony. Pathological hypotony in rabbits is generally defined as IOP < 5 mmHg [7]; even the maximum overestimation at 5 mmHg does not elevate the reading above this diagnostic threshold, thus avoiding false-negative outcomes. Therefore, while the device’s overestimation is clinically negligible for routine IOP monitoring, targeted correction is advised for studies requiring precise low-IOP quantification.

In the high IOP interval, the regression slope approaches 1 with a negative intercept, reflecting good overall agreement with the reference standard, albeit with a persistent slight systematic underestimation. This phenomenon parallels the findings of canine eye experimental studies: the TonoVet^®^ Plus and TonoVet^®^ tonometers exhibited regression slopes of 0.86 and 0.73, respectively, in the normal IOP interval—both significantly lower than their slopes in the high IOP interval (0.92 for TonoVet^®^ Plus and 0.90 for TonoVet^®^) [22]. This consistency across species-specific experiments suggests that current rebound tonometer algorithms generally exhibit measurement compression in the normal IOP interval, thereby limiting accuracy in this clinically critical interval. Although the iFalcon^TM^V100 tonometer demonstrates better goodness of fit in the high IOP interval (R² = 0.92) than in the normal IOP interval (R² = 0.87), its mean bias in the normal IOP interval is only 0.06 mmHg—far lower than the − 0.85 mmHg mean bias observed in the high IOP interval. This performance characteristic is clinically advantageous, given that normal IOP measurement constitutes the most common scenario in routine veterinary ophthalmology. Bland-Altman analysis further quantifies these interval-specific differences, showing a narrower random error range in the normal IOP interval (7.52 mmHg) compared to the high interval (9.54 mmHg).

Our findings are consistent with our initial hypothesis. The iFalcon^TM^V100 exhibited strong overall agreement with the reference sensor (R² = 0.97), indicating its suitability for assessing IOP in ex vivo rabbit eyes. As expected, accuracy was dependent on the IOP range: the device displayed minimal bias within the normal IOP range but systematically underestimated IOP at higher levels. Furthermore, measurements were unstable below 10 mmHg. These results highlight the importance of considering the IOP level when interpreting data obtained with rebound tonometers, particularly in experimental settings involving hypotony or elevated IOP.

### Limitations

Several limitations of the present study should be acknowledged. First, the sample size was limited to four eyes. While this sample size is consistent with the scope of many ex vivo validation studies in ophthalmic device research [38, 39], it inherently restricts the generalizability of our findings. Specifically, the limited sample precludes exploration of potential sources of biological variability—such as rabbit breed, age, and individual corneal phenotypic traits—that may influence device performance. Second, the ex vivo study design introduces inherent constraints. Postmortem changes in ocular tissue properties (e.g., altered corneal biomechanics, reduced tissue elasticity) and the inability to replicate physiological regulatory mechanisms of living eyes (e.g., dynamic tear film formation, homeostatic corneal hydration balance) may have amplified measurement errors [40]. This limitation is common to all ex vivo ophthalmic studies, as the ex vivo environment cannot fully mimic the in vivo ocular microenvironment. Third, methodological constraints were present: key corneal biomechanical parameters, including central corneal thickness (CCT) and corneal hydration state, were not measured or controlled for. Although slit-lamp examination confirmed the absence of gross structural abnormalities, inter-individual variations in these unmeasured variables likely contributed to the random error observed in the Bland-Altman analysis, limiting our ability to fully elucidate the sources of variability in device performance. Fourth, regarding device-specific limitations, the iFalcon^TM^V100 tonometer exhibited inherent instability in the extremely low IOP range (5–10 mmHg), indicating unreliability for consistent measurements in this interval. This range-dependent performance may be attributed to the technical characteristics of rebound tonometers, which rely on corneal deformation dynamics that are less predictable at extremely low IOP levels. Collectively, these limitations highlight the need for future in vivo studies with larger sample sizes and comprehensive assessment of corneal biomechanical parameters to validate the clinical utility of the iFalcon^TM^V100.

## Conclusions

The iFalcon^TM^V100 rebound tonometer demonstrates experimental utility in the ex vivo rabbit eye model: within the IOP range above 10 mmHg (covering the normal IOP range of rabbits, 10–25 mmHg), the device provides relatively accurate and reliable readings. For elevated IOP (> 25 mmHg), the device demonstrated reliable tracking capability. Nonetheless, we identified a consistent slight underestimation at these pressures. This bias must be accounted for during interpretation. Due to the observed instability and increased measurement failure rate at IOPs < 10 mmHg, the reliability of the iFalcon^TM^V100 is limited in this range. Users should therefore exercise caution when interpreting readings in this range, as the inherent measurement error is substantial.

## Data Availability

The data supporting the findings of this study are available from the corresponding author upon reasonable request.
